# Development of Conceptual Flexibility in Intuitive Biology: Effects of Environment and Experience

**DOI:** 10.3389/fpsyg.2020.537672

**Published:** 2020-09-16

**Authors:** Nicole Betz, John D. Coley

**Affiliations:** Department of Psychology, Northeastern University, Boston, MA, United States

**Keywords:** intuitive biology, ecological knowledge, conceptual flexibility, experience, salience

## Abstract

Living things can be classified in many ways, such as taxonomic similarity (lions and lynx), or shared ecological habitat (ducks and turtles). The present studies used card-sorting and triad tasks to explore developmental and experiential changes in *conceptual flexibility*–the ability to switch between taxonomic and ecological construals of living things–as well as two processes underlying conceptual flexibility: *salience* (i.e., the ease with which relations come to mind outside of contextual influences) and *availability* (i.e., the presence of relations in one’s mental space) of taxonomic and ecological relations. We were also interested in the extent to which salience and availability of taxonomic and ecological relations predicted inductive inferences. Participants were 452 six to ten-year-olds from urban, suburban, and rural communities in New England. Across two studies, taxonomic relations were overwhelmingly more salient than ecological relations, although salience of ecological relations was higher among children from rural environments (Study 1) and those who engaged in unstructured exploration of nature (Study 2). Availability of ecological relations, as well as conceptual flexibility, increased with age, and was higher among children living in more rural environments. Notably, salience, but not availability, of ecological relations predicted ecological inferences. These findings suggest that taxonomic categories (i.e., groups that share both perceptual similarities and rich underlying structure) are a salient way to organize intuitive biological knowledge and that, critically, environmental richness and relevant experience contribute to the salience and availability of ecological knowledge, and thereby, conceptual flexibility in biological thinking. More generally, they highlight important linkages between domain-specific knowledge and domain-general cognitive abilities.

## Introduction

The biological world is complex and multidimensional; living things can be interrelated—and therefore organized—in many different ways. For instance, *taxonomic relations* group living things into kinds based on morphological similarities, shared intrinsic features, or common membership in superordinate categories. Orthogonal to relationships based on intrinsic features, a large part of our knowledge about the biological world relates to extrinsically or thematically shared features. For example, organizing living things by *thematic* relations can describe ecological relationships such as a common habitat, or interactions such as predation. Both taxonomic and ecological relations, although far from exhausting potential relations among living things, are useful classifications, and both emphasize some types of commonalities at the cost of others.

Given that there are multiple ways to meaningfully organize plants and animals, what factors influence the relations that we use to group organisms or to make inductive generalizations about them? In the present studies, we used a card sorting task to explore developmental changes in the relative salience and availability of taxonomic and ecological relations among living things. We also examine the effects of two overarching types of active experiences—unstructured (e.g., exploring nature) and structured (e.g., going to zoos)—and passive experience (e.g., living in a rural area) thereon. Of particular interest is the development of the ability to organize the biological world through multiple frameworks–an ability likely to be related to both richness of biological experience (Shafto and Coley, 2003; Coley, 2012) and the development of more general executive functions (Diamond, 2013; Zaitchik et al., 2014).

This type of *conceptual flexibility* (Blaye et al., 2006) is critical in learning biological science as well as other STEM fields (Coulson et al., 1997; Hoehn and Finkelstein, 2018). In biology, one must be able to think about systems and organisms at a variety of different levels of analysis –the function of an organic molecule in a cell, the function of a cell in an organ, the function of the organ in a body, the function of a body in an ecosystem. Developing such flexible understanding is an important step toward learning to ‘think like a biologist.’ Thus, by investigating the developing understanding that organisms can be cross-classified in multiple ways, and the impact of active and passive experience thereof, we examine the development of conceptual systems that form an important basis for learning science. Further, this research evaluates the impact of ecologically valid experiences that impact the degree to which children come into the classroom with an ability to think flexibly about relations among organisms.

### Beyond a ‘Thematic to Taxonomic Shift’?

For many years, conventional wisdom among cognitive development researchers held that young children’s concepts are organized in terms of thematic relations, and that only later in development do they use taxonomic relations (e.g., Inhelder and Piaget, 1961/1999; Bruner et al., 1967; Smiley and Brown, 1979; see also Lin and Murphy, 2001 for a review). Thematic concepts were thought to be more accessible to young children because they are more concrete; the associated objects are perceived together and interact directly in salient ways. As they age, children begin to understand how objects are linked based on deeper or less obvious shared properties, which gives rise to taxonomic relations. Older children and adults were thought to primarily rely on purportedly more abstract and logical taxonomic relations, and less on allegedly primitive thematic relations. Based on this view, conceptual development was characterized as a shift from thematic to taxonomic relations (Smiley and Brown, 1979).

More recently, this “thematic-to-taxonomic” shift has been questioned in at least two ways. First, researchers consistently find that young children readily use taxonomic relations to sort objects and guide inferences (e.g., Scott et al., 1982; Gelman and Markman, 1986; Gelman and Coley, 1990; Waxman and Namy, 1997; Nguyen, 2007). For example, preschoolers expect that novel words (e.g., ‘See this *wem*? Find another one) refer to taxonomic rather than thematic relations (Markman and Hutchinson, 1984). Likewise, work on the development of intuitive biology has demonstrated an early preference for taxonomic relations for guiding biological *inductive inferences*, or projecting features believed to be true of one class to another related class (e.g., Coley and Vasilyeva, 2010). For example, children as young as 2.5 years of age rely on taxonomic relations (e.g., *birds*) when projecting potentially shared properties (e.g., *lives in a nest*; Gelman and Coley, 1990). Indeed, even 14–16 month old infants’ use of labels suggests sensitivity to the importance of membership in taxonomic categories to guide inferences (Keates and Graham, 2008; see also Ferry et al., 2010; Graham et al., 2016; Switzer and Graham, 2017). This and other work (for reviews, see Coley et al., 2002; Coley and Muratore, 2012) suggest that taxonomic relations based on intrinsic, inherent similarities can be more salient for young children than thematic relations. Together, these and other findings (e.g., Nguyen and Murphy, 2003; Coley et al., 2005; Thibaut et al., 2016) debunk the notions of the inaccessibility of taxonomic relationships at an early age, and a ubiquitous early preference for thematic relations in young children.

It’s important to note an ongoing debate among researchers in conceptual development on the nature of early category representations. The work reviewed above suggests that young children represent categories as richly structured conceptual representations, entailing assumptions of shared non-obvious features and inductive potential (e.g., Gelman and Coley, 1991; Gelman, 2003; Noyes and Keil, 2019). In contrast, other researchers characterize the process of category acquisition as a progression from simple perceptual groupings to more complex, abstract concepts (e.g., Sloutsky and Fisher, 2004; Sloutsky, 2010), and that early categories and inductions *primarily* stem from perceptual similarity (Sloutsky et al., 2007; Fisher, 2015). By this account, the early salience of “taxonomic” categories stems from shared perceptual features rather than rich abstract representations. However, for our purposes, this debate is largely tangential. Our primary goal is to contrast the salience of categories based on taxonomic, or intrinsic relations–be they perceptual or abstract–with the salience of categories based on thematic, or extrinsic relations.

In addition to evidence suggesting early salience of taxonomic relations, cognitive scientists also increasingly acknowledge the salience and potency of thematic relations for adults, especially among those with relevant expertise. Indeed, thematic relations can be as salient as taxonomic relations across a broad range of categorization and reasoning tasks. For example, adults often prefer to sort entities based on meaningful thematic relations (e.g., pairing *sheep* with *wool*) over taxonomic (e.g., pairing *sheep* with *goat*; Lin and Murphy, 2001). Further, thematic categories (e.g., breakfast foods) importantly guide people’s representation of food categories (Ross and Murphy, 1999). Moreover, expert categorization of plants and animals are more likely to be dominated by thematic relations than novices’ (e.g., López et al., 1997; Shafto and Coley, 2003), suggesting that salience of thematic relations may be characteristic of expert knowledge (Coley et al., 2005; Coley and Betz, 2018). These and other findings (Medin et al., 1997; Lin and Murphy, 2001; De Deyne et al., 2016) highlight the potency and use of thematic relations in refined conceptual systems, and the potential role of experience in augmenting the salience of thematic relations.

### Flexibility in Conceptual Relations

Rather than characterizing conceptual development as a shift from reliance on one type of relation to reliance on another, it seems more pertinent to acknowledge that children and adults flexibly use a range of conceptual relations to understand and predict their world (e.g., Deak, 2003; Blaye et al., 2007; Nguyen, 2007; Coley and Vasilyeva, 2010). By this account, individuals have access to multiple types of relations around which they can organize the same sets of entities; taxonomic and thematic relations are some examples of these organizational options. Flexibility in conceptual relations describes how the usefulness and subsequent recruitment of different conceptual relations might shift across contexts based on one’s knowledge. For example, both thematic and taxonomic relations can provide a rich inductive basis for different types of biological judgments. While taxonomic relations are more potent for making predictions about shared properties that are transmitted through genes, one type of thematic relation—ecological—is more potent for making predictions about shared properties that can be transmitted through contact, such as contagious diseases (e.g., Shafto and Coley, 2003; Coley and Vasilyeva, 2010; Coley, 2012). From this perspective, development involves an increase in the ability to flexibly use a range of different conceptual relations in a given domain. This ability is dependent on both domain-general executive functions (inhibitory control, cognitive flexibility) and domain-specific knowledge of relevant conceptual relations.

Although multiple relations might come to mind as *available* ways to organize entities, these relations often differ in their *salience* to different individuals, and therefore vary with respect to the likelihood that they will be used in any given situation (Shafto et al., 2007b). By “availability” we mean whether knowledge of particular conceptual relations is represented in semantic memory, or readily discernible given the information that is represented in semantic memory. Given that it is possible to hold multiple representations of relations among concepts in a given domain (e.g., Spiro, 1988), availability refers to which representations a particular individual actually holds. By “salience” we mean the ease with which knowledge of a particular type of conceptual relation comes to mind or is retrieved; different conceptual relations, even if all are available to an individual, can vary in their salience. The independence of these two ideas can be seen in the work of Ross and Murphy (1999) regarding food categories. They found that when given a food like *broccoli*, undergraduate participants readily and spontaneously generated both taxonomic (e.g., *vegetables*) and thematic (e.g., *dinner foods*) categories to which the food belonged, demonstrating that both types of categories are available. However, they also found that priming thematic categories (e.g., *breakfast foods*) increased the perceived similarity of thematic matches (e.g., *bagels* and *orange juice*) whereas priming taxonomic categories (e.g., *meat*) had no effect on the perceived similarity of taxonomic matches (e.g., *bacon* and *beef*), suggesting that taxonomic categories were already highly salient and therefore were unaffected by priming, whereas thematic categories were initially less salient and therefore priming increased their salience. Similarly, among students at an urban university, the ability to make thematic inferences about novel properties of animal species diminished under time pressure, whereas time pressure had no effect on taxonomic inferences (Shafto et al., 2007a), suggesting that although both types of relations were available, taxonomic relations among animals were more salient than thematic (i.e., ecological) relations in this population.

Both the availability and salience of conceptual relations seem to be influenced by knowledge and context (for reviews, see Coley et al., 2005; Shafto et al., 2007b). For example, undergraduates without marine expertise rely on taxonomic relations when making any generalizations about fish; in contrast, commercial fishermen only rely on taxonomic relations when reasoning about unknown novel properties (e.g., *has endo inside*), but used ecological relations to reason about relevant known properties, such as disease (Shafto and Coley, 2003). Therein, experts demonstrate greater conceptual flexibility, presumably due to the availability of a wider range of knowledge about relations among marine species (Shafto and Coley, 2003). This and other work (e.g., López et al., 1997; Medin et al., 1997; Bailenson et al., 2002; Ross et al., 2003; Medin et al., 2006) suggest that experience in nature may influence the flexible ability to reason about biology based on multiple types of relations.

Knowledge and context may also influence the development of conceptual flexibility in the domain of biology. Notably, context could refer not only to task constraints, but also to the information that is consistently present in one’s environment, such as the rurality of their home. For example, Coley (2012) examined inductive inferences in a forced-choice property projection task. Children in grades *K*−6 from urban, suburban, and rural communities were taught a novel property (i.e., *disease*, or *insides*) about one species (e.g., *cattail*), and asked which of two target species were more likely to share the property. One target species was related taxonomically but not ecologically (e.g., *dandelion*), whereas the other was related ecologically but not taxonomically (e.g., *frog*). Overall, the tendency to make taxonomic inferences about insides was similar among children across age, environment and experience. In contrast, the tendency to make ecological inferences about *disease* increased with age and was higher among children with direct unstructured experience in nature (e.g., unsupervised exploration) and among children who lived in more rural areas. Moreover, pronounced patterns of selective inference were evident earlier among children from rural communities. Six-year-olds from rural communities preferred to make ecological inferences about novel diseases and taxonomic inferences about novel insides; these patterns were not evident until later among children from suburban or urban communities. This suggests that children with more opportunities to interact directly with relatively intact ecosystems may demonstrate precocious conceptual flexibility with respect to inductive reasoning about plant and animal species.

If conceptual flexibility in the domain of biology emerges earlier in rural children, it would underline the importance of ambient environment and relevant experiences in the development of knowledge of conceptual relations. However, the evidence from Coley (2012) is indirect; children reasoning about *insides* preferred taxonomic inferences, whereas children reasoning about *disease* preferred ecological inferences, but the flexibility of individual children was not tested. Moreover, it does not pinpoint the source of the differences. There are at least two different ways in which young rural and urban children might differ in this respect: in terms of availability of conceptual relations, and in terms of salience. First, the differences in conceptual flexibility may be attributable to differences in *availability* of ecological relations (Shafto et al., 2007b). Unlike rural children. younger urban children may not understand how to meaningfully connect local organisms ecologically because they lack experience with these habitats. Indeed, modern suburban and urban children are less likely to learn about ecological relations through unstructured, exploratory play because rural children spend relatively more time in nature where they could informally observe which local animals cohabitate (e.g., Bunting and Cousins, 1985; Beach, 2003; Coley, 2012). If this is the case, younger urban children might rarely, if ever, organize living things based on shared ecology, whereas younger rural children might have such relations available. Alternatively, younger urban children may have both taxonomic and ecological relations available to them–i.e., possess the requisite knowledge of both taxonomic and ecological relations–but prefer taxonomic reasoning due to the relative *salience* of such relations over ecological. If this were the case, we would expect younger urban children to *be able to* organize living things based on shared ecology, but to *prefer* taxonomic relations as their initial response. Thus, to further explore conceptual flexibility of biological reasoning in young children, the current research investigates the developmental trajectory of both availability and salience of ecological and taxonomic relations, and the effects of experience and environment thereon. Beyond describing how children develop intuitive biological thinking, findings will enhance understanding of the underlying conceptual processes that contribute to conceptual flexibility.

### The Current Research

In the studies presented here, we address two sets of research questions. First, we examine developmental and experience-based changes in the *salience and availability of taxonomic and ecological relations* in children’s intuitive biological thinking. Specifically, we ask: To what extent are taxonomic and ecological relations among living things salient and available to children in grades *K*−6? How does the salience and availability of these relations change with development? How might children’s environment and experiences with nature influence the salience of taxonomic versus ecological relations? Do salience and availability of taxonomic and ecological relations predict patterns of inductive inference? Second, we probe *conceptual flexibility* in the domain of biology (i.e., the ability to shift between taxonomic and ecological thinking about living things). Specifically, we ask: To what extent are children able to demonstrate conceptual flexibility? How does conceptual flexibility change with development? How might children’s environment and experiences with nature influence conceptual flexibility?

To answer these questions, we conducted two studies wherein we sampled 452 children in *K*−6th grade from urban, suburban, and rural communities in New England. In Study 1, we used an unguided card sorting task to examine the relative salience of ecological and taxonomic relations among plants and animals. Children sorted pictures into groups, and explained their groupings. We predicted that ecological relations would be more salient for children from rural communities, who have more opportunities for experience with shared local ecological relations, and would also become more frequent with age.

In Study 2, we expanded on this methodology in several ways. We sampled a much larger group of children from a wide range of communities in Massachusetts. We asked children to perform two sorts of the same stimuli, thus eliciting information on what relations are *available* (second sort) as well as which relations are *salient* (first sort). This allowed us to directly assess conceptual flexibility by observing children’s ability to produce both taxonomic and ecological sorts. We also collected data on children’s nature-related experiences. This allowed us to investigate the degree to which a relevant feature of environment (i.e., population density of hometown) and direct experience may impact both the salience and availability of ecological and taxonomic relations. We predicted that both passive experience with nature that comes from living in a more rural environment, as well as direct experiences with nature would lead to increased salience and/or availability of ecological relations, and increased conceptual flexibility in the biological domain.

Finally, in order to test the implications of salience and availability of taxonomic and ecological relations for generalization, we examined relations between sorting responses and previously published data on patterns of inductive inference in the same sample of children (Coley, 2012). Of interest is whether the salience and/or availability of ecological relations in sorting would predict an increase in the flexible use of ecological relations when making inductions.

By investigating the development of the understanding that organisms can be cross-classified in multiple ways, and the impact of active and passive experience on this understanding, we are examining the development of conceptual systems that form an important basis for learning science, and evaluating the impact of relevant ecological experiences that would impact the degree to which children come into the classroom with an ability to think flexibly about relations among organisms.

## Study 1

In Study one, we investigated the relative salience of taxonomic and ecological relationships for 6- through 10-year-olds using an unguided card sorting task, and the effects of experience thereon. A relatively unconstrained way to examine the relative salience of taxonomic versus ecological relations is to present a set of stimuli in which both kinds of relations are present, and note which relations children use to spontaneously sort the stimuli. Thus, participants were asked to sort a set of 15 detailed color drawings of plants and animals that were designed to fall into orthogonal taxonomic and ecological groups. Of interest is the relative frequency of taxonomic versus ecological groupings and explanations in each population of children. If experience leads to increased salience of ecological relations, then the frequency of ecological groups and ecological explanations should be higher for children from rural communities than for children from urban communities.

### Method

#### Participants

One hundred and six children from kindergarten through 6th grade were recruited through elementary schools during the 2000–2001 school year. The vast majority of participants spontaneously sorted all the cards and provided explanations, but three participants were omitted for failure to perform the task (i.e., either did not sort all cards or did not provide explanations), yielding one hundred and three total cases. For our analyses, we classified children as rural or urban based on the population density of their town. Although not a direct measure of potentially relevant experience, children from less densely populated areas likely have increased opportunities to interact with plants and animals in relatively intact ecosystems. Fifty-five participants were from East Boston, which was classified as ‘urban’ (population density 12,166 people per square mile). Forty-nine participants were from one of two different rural communities: North Berwick, Maine (population density 112 people per square mile) and Plainfield, New Hampshire (population density 43 people per square mile). We also grouped participants by age based on their grade into “6-year-olds” (kindergarten; *N*_*rural*_ = 16, *N*_*urban*_ = 16), “8-year-olds” (second grade; *N*_*rural*_ = 17, *N*_*urban*_ = 14) and “10-year-olds” (fifth grade; *N*_*rural*_ = 16, *N*_*urban*_ = 25). These age groups are interesting for several reasons. Kindergartners are just beginning formal education, and as such are unlikely to have had extended exposure to formal science curricula, so their responses are likely to represent informally acquired folk knowledge. Moreover, between the ages of 6 and 10, important developmental changes occur in biological reasoning (see Carey, 1985).

#### Materials and Design

Stimuli consisted of fifteen 3 in. × 5 in. (7.6 cm × 12.7 cm) laminated cards. Each card contained a realistic color drawing of a local species. Species were chosen to fill a 5 (taxonomic class: *mammals*, *birds*, *insects*, *trees*, and *plants*) × 3 (habitat: *meadow*, *forest*, and *wetland*) matrix (see Table 1). Although we acknowledge that ‘trees’ does not reflect an evolutionarily coherent group of organisms, it is a highly salient and nearly universal category in folk taxonomy distinction between trees and plants is a highly robust folk taxonomic distinction (e.g., Brown, 1984; Berlin, 1999; Coley and Muratore, 2012). Notably, the included stimuli from taxonomic classes also shared many perceptual similarities; we did not include sets of taxonomic stimuli that featured highly perceptually variable members (e.g., a whale and a fox both as mammals).

**TABLE 1:** Organisms depicted in sorting task, Study 1.

	Ecological (habitat) category		
	
Taxonomic category	Forest	Meadow	Wetland
Mammal	Red squirrel	Woodchuck	Beaver
Bird	Screech owl	Meadowlark	Loon
Insect	Gypsy moth	Firefly	Dragonfly
Tree	White pine	Paper Birch	Black Willow
Plant	Fern	Milkweed	Cattails

#### Procedure

Pictures were arrayed on a table in a random order, and participants were asked to “Put together the things that go together.” After sorting all of the pictures into groups, children were asked to explain why they formed each group. The experimenter noted the cards in each group, and wrote down the child’s explanation for each group.

### Results

#### Data Coding

Of particular interest in this study were differences in ecological and taxonomic conceptual structures across age and population density. We assessed the degree to which children’s sorts reflected taxonomic and ecological relations in two ways. First, we looked at which organisms each child actually placed in the same group. Second, we also examined the *explanations* that each child provided for the groups they formed. These are explained in more detail below.

##### Groups

For each child, we noted the number of groups formed. We also quantified the content of children’s groupings by first counting the number of taxonomic pairs and ecological pairs, and then calculating percentage based on the total number of pairs within each child’s sort, akin to the strategy used by Krieter et al. (2016). A taxonomic pair consisted of two species from the same taxonomic category being grouped together (e.g., *woodchuck-beaver or fern-milkweed*). An ecological pair consisted of two species from the same ecological category being grouped together (e.g., *loon-dragonfly or firefly-paper birch*). This calculation allowed us to determine the extent to which children’s groups corresponded to our *a priori* assignment of species into taxonomic and ecological groups.

##### Explanations

Children’s explanations for their groupings were transcribed and coded independently for content by 3–4 trained coders using a constant comparative method (Glaser and Strauss, 1967; Boeije, 2002). Rare cases of disagreement were resolved via discussion. Explanations for each grouping were initially coded into four categories. *Taxonomic* explanations were based on common membership in named categories (e.g., “bugs,” “animals,” and “trees”). *Ecological explanations* were based on interactions among organisms or between organisms and their environment (e.g., “squirrels like trees,” “water animals,” and “birds live in trees”). *Appearance* explanations were based on surface attributes or properties such as size, color, shape, or appendages (e.g., “feathers,” “hair,” and “these look the same”). Finally, uninterpretable or non-sensical or irrelevant explanations were coded as *Other*. Except for the last one, categories were not mutually exclusive, and responses could fall into multiple categories. Inclusion of explanations allowed us to capture when children’s rationales for pairings diverged from our *a priori* predictions. For example, children may have paired screech owl, meadowlark, and loon together, and explained that “they all live in trees.” Although this would be coded as three taxonomic pairs, the explanation highlights the observation that they all share an extrinsic relation to trees, which we would consider ecological. Thus, examining explanations as well as groups gives us a fuller picture of children’s subjective basis for categorizing these organisms.

#### Performance on Sorting Task

Performance on the sorting task was taken as an index of the relative salience of different relations among stimulus species. Of interest are the relative frequency with which children paired ecologically and taxonomically related species, the content of children’s explanations for their groupings, and differences in these measures as a function of age and environment.

##### Groups

**FIGURE 1:**
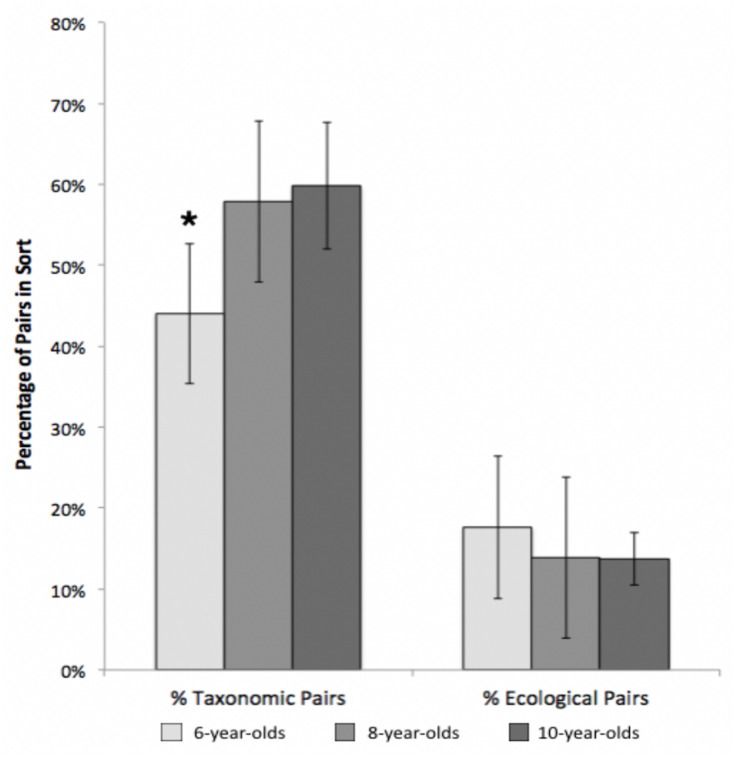
Age differences in percentages of taxonomic and ecological pairs, Study 1. Error bars represent 95% confidence intervals. *p < 0.001.

On average, children sorted the 15 cards into 4.4 groups (*SD* = 1.54). To investigate the effects of age and environment on the frequency with which taxonomic and ecological pairs were grouped together, we conducted a 2 (population group: urban, rural) by 3 (age group: 6-, 8-, and 10-year-olds) by 2 (percentage pair: taxonomic, ecological) mixed ANOVA. Overall, sorts were comprised of a higher percentage of taxonomic pairs (*M* = 55%) than ecological pairs (*M* = 15%), *F*(1,98) = 137.61, *p* <0.001, eta^2^ = 0.58. We also found that participant age significantly influenced the sorted groups, as demonstrated by an age x pair type interaction ([F(1, 98) = 3.55, *p* = 0.033, eta^2^ = 0.07)]. Specifically, 6-year-olds created fewer taxonomic pairs than 8-year-olds or 10-year-olds, who did not differ (Tukey HSD *p* <0.05), whereas age had no effect on number of ecological pairs (see Figure 1). We found no significant differences between children from rural or urban communities on number of taxonomic or ecological pairs produced.

##### Explanations

Each child was given 2 scores, corresponding to the proportion of groups justified with *taxonomic*, or *ecological* explanations. Explanations based on appearance were relatively rare (*M* = 10%), and did not differ in frequency for urban versus rural children (*t* = 1.045, *p* = 0.299). Although explanations corresponded somewhat to pairs, a lack of significant correlation between frequency of pairs and explanations (see Table 2) suggests that these two metrics might indeed capture different aspects of children’s sorting behaviors.

**TABLE 2:** Correlations between percentage pairs and explanations, Study 1.

	*Explanations*	
	
	Taxonomic	Ecological
**Pairs**		
Taxonomic	0.166^+^	–0.071
Ecological	–0.058	0.128

*Table reports Spearman’s correlation coefficients. ^+^p < 0.10.*

**FIGURE 2:**
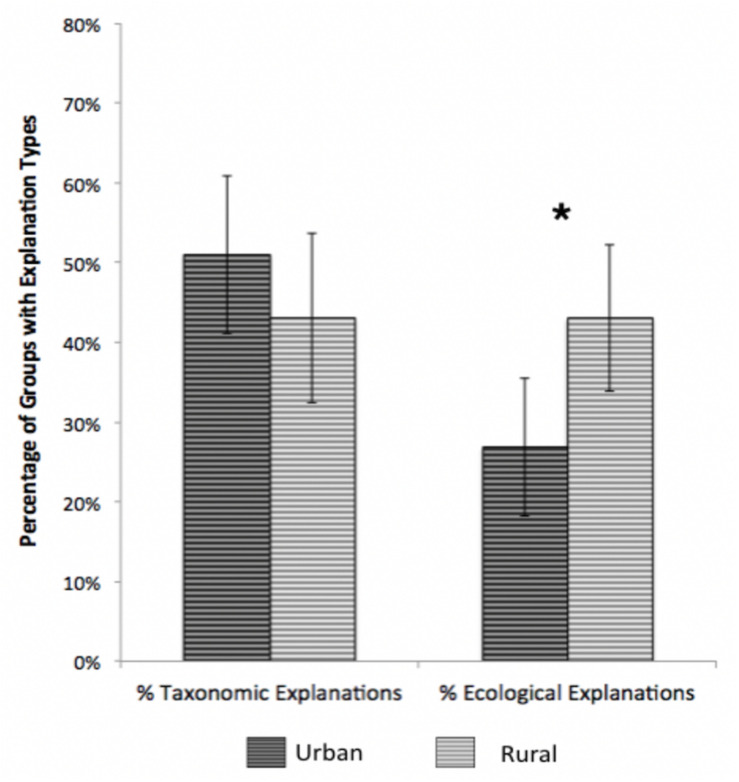
Population group differences in percentages of taxonomic and ecological explanations of sorted groups, Study 1. Error bars represent 95% confidence intervals. *p < 0.001.

To investigate the effects of age and environment on children’s explanations for their groups, we conducted a 2 (Population Group: Urban and Rural) × 3 (Age Group: 6-, 8-, and 10-year-olds) × 2 (explanation: Taxonomic and Ecological) mixed ANOVA, with population group and age as between-subjects variables. Overall, percentage of taxonomic explanations (*M* = 47%) were higher than those for ecological (*M* = 35%), *F*(1,98) = 4.09, *p* = 0.046, η^2^ = 0.04. In contrast to findings for groups, children from urban and rural communities showed different patterns of explanations for their groupings, as evidenced by a population group × explanation interaction, *F*(1,98) = 4.16, *p* = 0.04, η^2^ = 0.04 (see Figure 2).

Simple effects analyses revealed that for urban children, taxonomic explanations were more frequent than ecological explanations, *t*(54) = 3.04, *p* = 0.004, Cohen’s *d* = 0.41. In contrast, for rural children, taxonomic and ecological explanations were equally frequent. Finally, *t*-tests directly comparing relative frequencies of each explanation in the two populations revealed that rural and urban children did not differ in the frequency of taxonomic explanations, but rural children produced significantly more ecological explanations than urban children, [*t*(102) = 2.55, *p* = 0.012, *d* = 0.50]. No effects of age on explanations were observed.

### Discussion

These results suggest that taxonomic relations are highly salient to children from both urban and rural communities; children at all ages showed an overwhelming preference to group together species from the same taxonomic class (e.g., *mammals* and *trees*) rather than from the same ecological habitat (e.g., *wetlands species*). Taxonomic explanations were also relatively frequent in both population groups at all ages; almost half of all groupings were explained on taxonomic grounds. Although there was an increase in taxonomic pairs with development, taxonomic relationships still dominated the 6-year-olds’ sorts and explanations. Thus, these results reinforce previous findings showing the importance of taxonomic relations in organizing biological knowledge (e.g., Coley et al., 2002; Coley and Vasilyeva, 2010; Coley and Muratore, 2012) with the caveat that taxonomic relationships become even more salient between the ages of six and eight.

Because the taxonomic stimuli were more perceptually similar than the ecological ones, a potential alternate explanation for the salience of taxonomic relations within our sample is these perceptual similarities. Although it is clear that taxonomic relations were quite salient to children—they not only grouped together taxonomic relations, but also used taxonomic explanations (e.g., “these are birds”) to justify their categories instead of explaining groups based on shared appearances—it is possible that the perceptual similarities may have cued the children toward fixating on shared taxonomic relations among the stimuli set. Indeed, the perceptual similarities that occur within taxonomic groups likely contribute to their salience. Importantly, in the real world, categories that share rich internal underlying structure also tend to share perceptual and morphological similarities; bats and whales are the exception, not the rule. Likewise, the taxonomic categories we used here, because they were drawn from reality, included members who shared perceptual as well as deep similarities. As such, although we demonstrate a clear early preference for taxonomic categories, we cannot determine whether this early preference was based on assumptions of shared rich structure, perceptual similarity, or some combination thereof.

Critically, in addition to the clear importance of taxonomic relations, an examination of children’s explanations for their groupings showed that, as predicted, ecological relations were more commonly used to justify groupings among children from rural communities than those from urban communities. Indeed, among children from rural communities such explanations were as frequent as taxonomic explanations, suggesting an important influence of ecological thinking and awareness of ecological relations among rural children not seen for urban children. These results are remarkably consistent with results from studies that link informal experience with nature to increased use of ecological relations among species (e.g., Boster and Johnson, 1989; López et al., 1997; Medin et al., 1997; Shafto and Coley, 2003; Ross et al., 2003; Coley, 2012). This difference likely arises from the richer ecological experience of children living in more rural areas, resulting in knowledge of ecological relations which are not perceptually obvious. We investigate this in more detail in Study Two.

## Study 2

Study one demonstrated that children living in a rural environment were no more likely to form ecological groupings, but were more likely to explain their groupings in ecological terms than children living in an urban environment. This suggests a relationship between environment and salience of ecological relations in children, but leaves unanswered the question of availability of ecological knowledge among children for whom taxonomic relations are salient. In Study 2, we tested a much larger sample of children from a wider range of communities and ages than in Study 1, and sought to replicate and extend the results of Study 1 in several ways.

While the more salient mode of organization (i.e., taxonomic) was the same across rural and urban populations, a single sorting task might underestimate differences in the availability of ecological knowledge across populations. In Study 2, we therefore asked participants to complete two sorts of the same cards, with specific direction to sort the cards in two different ways. While we expected taxonomic relationships to dominate the first sort again, we hypothesized that performance in the second sort would reflect the availability of ecological relationships after highly salient taxonomic relations were utilized on the first sort. If experience with nature fosters availability of ecological relations, we should find that rural children are more likely to sort species on the basis of ecological relations on a second sort. Moreover, this approach allows us to investigate the development of–and environmental differences in–conceptual flexibility surrounding reasoning about living things, by comparing the basis of children’s first and second sorts. Of particular interest was the extent to which children were able to organize the cards based on the two orthogonal dimensions of interest (e.g., Zelazo, 2006; Deák and Wiseheart, 2015).

Further, although the results from Study 1 document a marked difference in explanations for groupings between urban and rural children, they do not pinpoint which aspects of living in a rural environment encourage understanding of ecological relationships. Therefore, in Study 2 we included in-depth activities surveys (inquiring about relevant experiences with plants and animals) and examined the impact of activities in nature on sorting task performance. Specifically, we examine two broad types of potentially relevant experiences: unstructured experiences in nature (e.g., nature exploration, hiking), and learning about plants and animals in structured environments intended to transmit information, albeit informally (e.g., going to zoos or aquariums). In this sample, rural children were more likely to report unstructured activities, but rural, suburban and urban children did not differ in their engagement in structured activities (Coley, 2012). Indeed, children who attend these types of formal educational experiences (i.e., immersive zoo camps) demonstrate marked improvements in their ability to organize animals into taxonomic groups (Unger and Fisher, 2019).

Moreover, we addressed a key methodological concerns with Study 1 by revising our stimuli set. In Study 1, we had a larger proportion of possible taxonomic groups, which could have increased the relative salience of taxonomic relations for our participants. The revised stimuli set in Study 2 therefore contains equal numbers of *a priori* ecological and taxonomic groups.

Additionally, to investigate relations between the distinct but related conceptual processes of intuitive biological categorization and inductive reasoning, we examined whether the relative salience and availability of ecological and taxonomic relations during the sorting task predicted children’s tendency to draw inductive inferences based on taxonomic or ecological relations.

### Method

All of the data reported in Study 2 were collected as a part of a larger study. The inductive inference results, and their relations to the activities measures, are reported in detail in Coley (2012). Here we focus on the sorting data, which have not been reported elsewhere, and relations between the children’s sorting and their activities and inferences.

#### Participants

Three hundred sixty-two children from kindergarten through sixth grade were recruited through elementary schools and after-school programs from 30 communities in Massachusetts. Data were collected between 2004 and 2007. The vast majority of participants spontaneously sorted all of the cards into groups. Fourteen participants were excluded from analysis for failing to sort all of the cards on either the first or second sort, resulting in a final sample of 348. Sampled communities ranged in size from 9 people per square mile to 15,400 people per square mile, with samples from rural (*N* = 112), suburban (*N* = 122), and urban (*N* = 118) communities. We also sampled children ranging from 4.5 to 12.75 years of age, including samples from grades *K*−1 (*N* = 117), grades 2–3 (*N* = 125) and grades 4–6 (*N* = 106). Children were roughly 60% girls and 40% boys; this distribution held in each subgroup.

#### Materials, Design, and Procedure

##### Sorting task

Stimuli consisted of nine 3 in. × 5 in. (7.6 cm × 12.7 cm) laminated cards. Each card contained a realistic color drawing of a local species. Species were chosen to fill a 3 (taxonomic class: *bird*, *invertebrate*, and *plant*) × 3 (habitat: *meadow*, *forest*, and *wetland*) matrix. Items are described in Table 3. We cut down on the number of cards included in this task for two reasons. First, a smaller number of cards would be less of a burden for the children to sort twice. Second, fewer taxonomic relationships allowed us to have the same number of possible taxonomic and ecological pairs, in case the presence of additional taxonomic groups in the sorting array increased the salience of taxonomic over ecological groups. As in Study 1, the stimuli within taxonomic categories did share more perceptual similarities than the stimuli within ecological categories.

**TABLE 3:** Pictures used in sorting task, Study 2.

	Ecological category		
	
Taxonomic category	Forest	Meadow	Wetland
Bird	Woodpecker	Blue jay	Duck
Invertebrate	Termite	Grasshopper	Slug
Plant	Pine tree	Milkweed	Cattails

Children were interviewed individually in a quiet area at their school or after-school program. The pictures were arrayed on a table in a random order, and participants were instructed to “Put together the things that go together.” After they sorted the cards, they were asked to explain why they had made each group. Then the cards were shuffled and spread out again and the children were asked if they could put the cards together in a new way, and then to explain their new groupings. The experimenter noted the groups for each of the two sorts, and transcribed children’s explanations.

##### Triad task

Stimuli consisted of 32 8.5 in. × 11 in. laminated cards. Each card featured three realistic color drawings of plants or animals: one premise species and two target species. The premise species was alone at the top of the card and the two target species were equidistant from the premise and located at the bottom of the card. One target was taxonomically related to the premise, but ecologically unrelated while the other target was ecologically related to the premise, but taxonomically unrelated. See Table 4 for a list of sample triad stimuli, and see Coley (2012) for full details on the triad task.

**TABLE 4:** Sample items used in induction task, Study 2.

Premise category	Taxonomic match	Ecological match
Oak tree	Grass	Squirrel
Cattail	Dandelion	Beaver
Herring	Clownfish	Penguin

Participants were told that a blank property was true of the premise category and asked which of the two target categories would share that property. Half of the participants were told that the target category “has stuff inside called *andro*.” This was meant to represent an internal property that we predicted would be understood to be anatomical. The other half of participants were told that the target category “has a disease called *andro*.” Blank property names changed across each trial.

##### Activities survey

Prior to the experimental task, each child was given a brief survey about their activities. Within the survey, children were asked questions about their hobbies and interests, with follow-up questions and encouragement to solicit additional information. The survey had questions about hobbies and activities that could have implications for intuitive biology, including pet ownership, gardening, zoos, parks, aquariums, camping, hiking, hunting, and fishing.

### Results

#### Data Coding

##### Sorting task

Each child’s performance on both Sort 1 and Sort 2 yielded two scores: first, the percentage of taxonomic pairs (e.g., woodpecker + blue jay) and ecological pairs (e.g., duck + cattails) out of all pairs produced by the child, and second, the percentage of groups justified by taxonomic and ecological relations (coded as in Study 1). As in Study 1, the percentage of pairs reflected the children’s adherence to our predicted taxonomic and ecological categories, while the percentage of justifications reflected the children’s perceptions of their groupings. Percentage of taxonomic and ecological pairs were calculated in the same way as in Study 1. However, we used a more comprehensive coding approach for explanations in Study 2. In general, performance on the initial sort was taken as an index of the relative salience of different relations among stimulus species. Performance on the second sort was taken as an index of the availability of children’s knowledge of relations among species after the initially salient relations had been identified.

Children’s explanations were again transcribed and coded independently for content by 3–4 trained coders using a constant comparative method (Glaser and Strauss, 1967; Boeije, 2002). Rare cases of disagreement were resolved via discussion. Explanations for each grouping were initially coded into the following categories, adapted from Coley and Vasilyeva (2010: (t) taxonomy, (e) general interaction, (e) diet interaction, (e) habitat interaction, (e) similar habitat, (e) similar diet, (n) similar behavior, (n) similar appearance, (n) general similarity, and (n) other. These original codes were thereafter reduced into three dimensions: taxonomic (t), ecological (e), and neither (n). See Supplementary Table 1 for detailed explanations of dimension reduction, example codes, and prevalence of each subtype of explanation.

##### Conceptual flexibility

We also created two indices of conceptual flexibility that assessed changes in pairs and in explanations across the two sorts. For pairs, we computed the absolute difference in the percentage of ecological pairs between sort one and sort two, and in the percentage of taxonomic pairs between sort one and sort two. We then averaged the two differences to yield an index of the degree to which responses on the two sorts differed. We also computed an analogous score for changes in ecological and taxonomic explanations. In both cases, higher values represent larger changes in the relative use of taxonomic or ecological relations from sort 1 to sort 2, which we take as evidence of conceptual flexibility. Although there are many ways that flexibility might be quantified, we used this approach to capture the degree to which children spontaneously switched the organizing framework of their sorts from one conceptual dimension of interest to the other.

##### Activities survey

Interpretable responses to all activities questions were obtained from 252 participants; these were fairly uniformly distributed between urban (*N* = 82), suburban (*N* = 72), and rural (*N* = 98) participants. Children’s responses to the activities survey were coded into 23 categories (14 developed to capture open-ended responses, and another nine activities that were specifically queried). These in turn were reduced to 10 activity categories via factor analysis (*Aquarium and Parks*, *Pets and Houseplants*, *Reading and Writing*, *Hunting and Hiking*, *TV and Toys*, *Sports*, *Outdoors and Fishing*, *Video and Board Games*, *Exploring Nature*, and *Animals and Zoos*). For details, see Coley (2012). For purposes of this study, we focused on activity types that were of specific interest to informal biological education: Unstructured Activities (indexed by averaging scores on the *hunting and hiking*, *outdoors and fishing*, and *exploring nature* factors) and Structured Activities (indexed by averaging scores on *aquarium and parks* and *animals and zoos* factors).

#### Salience and Availability of Taxonomic and Ecological Relations

In this section we investigate the salience and availability of taxonomic and ecological relations by examining children’s groupings, and explanations for those groupings, on both the first sort (which we take to index *salient* relations) and the second sort (reflecting knowledge of relations that may be *available* but not highly salient). We also explore the degree to which salience are availability of taxonomic and ecological relations are predicted by activities and environment, and how the salience and availability of these relations predict children’s inductive inferences.

##### What kinds of groupings did children form?

**FIGURE 3:**
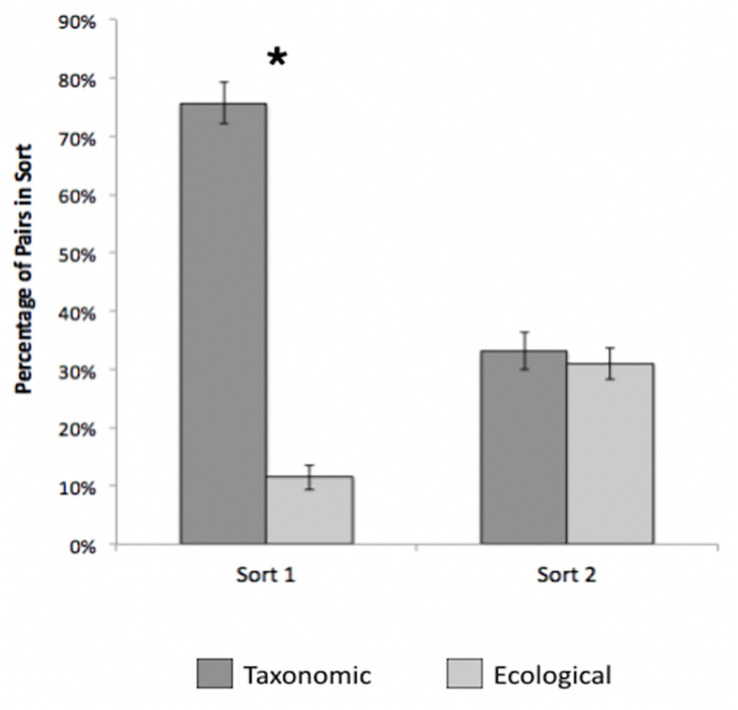
Percentage of taxonomic and ecological pairs across sorts 1 and 2, Study 2. Error bars represent 95% confidence intervals. *p < 0.001.

To examine the content of children’s groupings, we compared the relative frequency of taxonomic and ecological pairs for the first and second sort. On average, children sorted the nine cards into 3.5 groups on the first sort (*SD* = 0.82), and taxonomic pairs (*M* = 76%) represented a higher percentage of the sorts than ecological (*M* = 11%) pairs, [*t*(346) = 23.01, *p* < 0.001, *d* = 1.24, see Figure 3]. Children formed more groups on the second sort (*M* = 3.6, *SD* = 1.06) than on the first sort, although the effect was small [*t*(347) = 3.12, *p* = 0.002, *d* = 0.17]. In contrast to the first sort, taxonomic (*M* = 33%) and ecological pairs (*M* = 31%) were equally common on the second sort, [*t*(334) = 0.78, *p* = 0.44, *d* = 0.04, see Figure 3]. Proportion of taxonomic pairs declined across the two sorts [*t*(344) = 16.69, *p* < 0.001, *d* = 0.90], while the proportion of ecological pairs increased [*t*(343) = 10.89, *p* < 0.001, *d* = 0.59]. This suggests that taxonomic relations were highly salient to most children, but some switched to more ecologically based groupings on their second sort, indicating that ecological relations were nevertheless available.

##### What factors predicted children’s groupings?

To examine which factors predicted children’s groupings, we conducted multiple regression analyses utilizing age (in days), population density (people per square mile in child’s town), and reported involvement in structured and unstructured activities to predict the number of taxonomic and ecological pairs separately for sort 1 and sort 2. Because of the large range of population density values in our sample (22–15,400), we log-transformed these values. Results of these analyses are summarized in Table 5.

**TABLE 5:** Factors predicting groupings on sorts 1 and 2.

	Sort 1		Sort 2	
		
	Taxonomic	Ecological	Taxonomic	Ecological
	pairs	pairs	pairs	pairs
*R*^2^	0.038^+^	0.021	0.018	0.023
Age	0.198**	−0.139*	0.010	0.064
Population density	–0.016	–0.041	0.075	−0.143*
Structured activities	–0.043	0.035	–0.032	–0.001
Unstructured activities	–0.067	0.025	0.122^+^	–0.047

Values for age, population density, structured activities, and unstructured activities represent standardized regression coefficients. **p < 0.01, *p < 0.05, ^+^p < 0.10.

###### First sort

For taxonomic pairs, the overall regression approached significance, but the only significant predictor of performance was age, with older children’s sorts featuring higher proportions of taxonomic pairs. For ecological pairs, the overall regression was not significant, although age again significantly predicted performance. In contrast with taxonomic pairs, younger children’s sorts had higher proportions of ecological pairs. This suggests that the salience of taxonomic relations increased with age, and the salience of ecological relations decreased with age.

###### Second sort

For taxonomic pairs on the second sort, the overall regression did not explain significant variance, and no predictors explained significant variance. Nor was the overall regression significant for ecological pairs, although population density explained significant variance. Specifically, proportion of ecological pairs on the second sort decreased with population density (i.e., ecological pairs were more common for children from more rural areas), suggesting that ecological relations became increasingly available for children living in more rural environments.

##### How did children explain their sorts?

**FIGURE 4:**
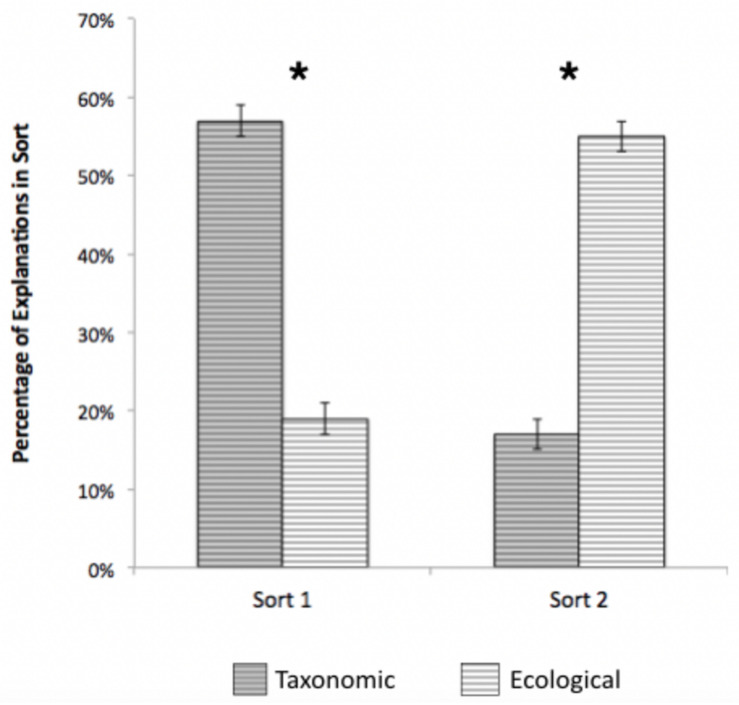
Percentage of taxonomic and ecological explanations across sorts 1 and 2, Study 2. Error bars represent 95% confidence intervals. *p < 0.001.

Explanations for groupings on the first sort, like the pairs, were overwhelmingly taxonomic (see Figure 4). Paired-samples *t*-tests revealed that taxonomic explanations (*M* = 59%) were significantly more common than ecological (*M* = 19%); *t*(347) = 11.476, *p* < 0.001, *d* = 0.62. In contrast, ecological explanations (*M* = 55%) were more common than taxonomic (*M* = 17%) on the second sort; *t*(346) = 12.19, *p* < 0.001, *d* = 0.58. When comparing explanations across the two sorts, the percentage of taxonomic explanations significantly declined [*t*(346) = 16.38, *p* < 0.001, *d* = 0.88], and the percentage of ecological explanations significantly increased [*t*(343) = 10.89, *p* < 0.001, *d* = 0.59]. This is consistent with the findings for pairs presented above; children tended to explain their initial groupings based on taxonomic relations, but explained their second sorting based on ecological relations, suggesting that taxonomic relations were highly salient, but ecological relations were nevertheless available.

##### What factors predicted children’s explanations?

To examine which factors predicted children’s explanations, we again conducted multiple regression analyses utilizing age (in days), population density (people per square mile in town, log transformed), and scores on structured and unstructured experience to predict the relative frequency of taxonomic and ecological explanations for sort 1 and sort 2. Results of these analyses are summarized in Table 6.

**TABLE 6:** Factors predicting explanations on Sorts 1 and 2.

	Sort 1		Sort 2	
		
	Taxonomic	Ecological	Taxonomic	Ecological
	explanations	explanations	explanations	explanations
*R*^2^	0.135***	0.032	0.034^+^	0.060**
Age	0.361***	–0.066	0.096	0.154*
Population density	–0.098	–0.042	−0.121 +	−0.153*
Structured experience	–0.038	0.083	–0.102	0.090
Unstructured experience	–0.057	0.147*	–0.001	−0.123^+^

Values for age, population density, structured activities, and unstructured activities represent standardized regression coefficients. ***p < 0.001, **p < 0.01, *p < 0.05, +p < 0.10.

###### First sort

For taxonomic explanations, the overall regression explained significant variance; the proportion of taxonomic explanations increased with age, suggesting that the salience of taxonomic relations increased with age but was unrelated to activities. For ecological explanations, the overall regression did not explain significant variance, but the proportion of ecological explanations increased with unstructured nature activities, suggesting that ecological relations were increasingly salient among children who engaged in one type of relevant experience—unstructured nature exploration.

###### Second sort

For taxonomic explanations, the overall regression did not reach significance and no factors predicted significant variance. For ecological explanations, the regression was significant; ecological explanations increased with age, and decreased with population density (i.e., were more common among children from more rural communities), suggesting that these factors impacted the availability if not the salience of ecological relations.

##### The relationship between explanations and groups

As in Study 1, the explanations and groups provided two different types of metrics of performance—adherence to our *a priori* taxonomic and ecological relations versus children’s reliance on taxonomic and ecological relations. In contrast to Study 1, we find strong positive correlations between taxonomic and ecological pairs and explanations within each of the sorts (see Table 7), a testament to the efficacy of our updated coding system and stimuli. The explanations and pairs in Study 2 therefore seem to converge quite highly. Further, we see a repeated negative relationship between taxonomic and ecological, suggesting that children are emphasizing one type of relation at the expense of the other.

**TABLE 7:** Correlations between percentage pairs and explanations, Study 2.

	Explanations, sort 1			Explanations, sort 2	
			
	Taxonomic	Ecological		Taxonomic	Ecological
**Pairs, Sort 1**			**Pairs Sort 2**		
Taxonomic	0.720***	−0.730***	Taxonomic	0.472**	−0.542**
Ecological	−0.656***	0.698***	Ecological	−0.336***	0.480***

Table reports Spearman’s correlation coefficients. ***p < 0.001, **p < 0.01.

##### How is performance on the sorting task related to children’s inductive inferences?

To examine relations between children’s sorting and their inductive inferences, we ran separate regressions using children’s first and second sort taxonomic and ecological pairs and explanations to predict their proportion of ecological inferences on the triad induction task (i.e., the percentage of trials for which a child projected the novel property to the ecological target rather than the taxonomic target). Each measure of performance was entered into a separate regression, along with age and log population density as control variables (both of which have been linked to ecological inferences, Coley, 2012). Results are reported in Table 8, and see Supplementary Tables 2, 3 for results from specific properties within the triad task.

**TABLE 8:** Relations between indices of performance on sorting task and preferences for ecological inferences in the triad induction task.

Sort	Relation type	Performance index	Standardized regression
			coefficient
1	Ecological	% Pairs	0.137*
		% Explanations	0.116*
	Taxonomic	% Pairs	−0.138*
		% Explanations	–0.063
2	Ecological	% Pairs	–0.011
		% Explanations	0.058
	Taxonomic	% Pairs	0.085
		% Explanations	0.029

*p < 0.05.

Regressions revealed several significant predictors of ecological inferences. Notably, salience of ecological relations, in terms of proportion of both ecological pairs and ecological explanations on sort 1, predicted frequency of ecological inductive inferences. Further, the proportion of taxonomic pairs in sort 1 negatively predicted frequency ecological inferences. No index of performance on sort 2 was related to ecological inferences. Overall, these findings suggest that the tendency to base inferences on ecological relations in the induction task was closely tied to the salience of ecological relations in the sorting task.

##### What factors predicted conceptual flexibility?

In this study, conceptual flexibility reflects the extent to which children changed between taxonomic and ecological organizational frameworks across their two sorts. In other words, we were primarily interested in the degree to which children produced different kinds of sorts—i.e., changed the underlying dimension along which they sorted organisms—rather than simply producing different groups. We therefore quantified conceptual flexibility as change (regardless of direction) in the percentage of taxonomic and ecological pairs, and change in the percentage of taxonomic and ecological explanations, between sort 1 and sort 2. Higher scores reflect more change from the first to second sort, and hence more flexibility. The two sorts provide qualitatively different information, with the first sort assessing the most salient relations and the second sort assessing additional relations that are available after the most salient ones were taken away. To the extent that children tended to rely on one underlying dimension for each sort, our approach could quantify the degree to which children were able to change this basis of their responses across sorts. This analysis was justified based on preliminary assessments of the data that revealed that sorts most often were dominated by either taxonomic or ecological relations, rather than split in between both types of relations (see Supplementary Figures 1–4; this effect is also highlighted by the negative correlations between ecological and taxonomic pairs and explanations presented in Table 7).

To examine which factors predicted flexible use of conceptual relations, we computed two multiple regression analyses utilizing age (in days), log population density (people per square mile in town), and structured and unstructured activity scores to predict conceptual flexibility with respect to pairs and explanations, respectively. Results are summarized in Table 9.

**TABLE 9:** Factors predicting conceptual flexibility scores for pairs and explanations.

	Flexibility index	
	
	Pairs	Explanations
*R*^2^	0.123***	0.144***
Age	0.313***	0.373***
Population density	−0.131*	–0.089
Structured activities	−0.143*	–0.006
Unstructured activities	–0.065	–0.043

Values for age, population density, structured activities, and unstructured activities represent standardized regression coefficients. ***p < 0.001, *p < 0.05.

The regression models explained significant variance in children’s conceptual flexibility with respect to both pairs and explanations. Conceptual flexibility regarding pairs increased with age and decreased with population density (i.e., children from more rural environments showed more flexibility). Surprisingly, children who reported more structured activities like aquariums and zoos also showed less conceptual flexibility. Conceptual flexibility regarding explanations increased with age, but was unrelated to environment or experience.

### Discussion

Results from the initial sort in Study 2 replicated the findings from Study 1 with a different population and stimulus set. Participants were much more likely to pair species on the basis of taxonomic than ecological relatedness; this tendency increased with age and held constant regardless of children’s environment. As in Study 1, taxonomically related stimuli were also perceptual more similar; as such, we cannot determine whether this early preference was based on assumptions of shared rich structure, perceptual similarity, or both.

Further, we again found some evidence of an effect of experience on explanations for sorts, if not sorting *per se*. In Study 1, ecological explanations were more frequent among children from rural environments, whereas in Study 2, ecological explanations increased with unstructured experiences in nature. Moreover, Study 2 revealed that knowledge of ecological relations, if not overly *salient*, is nevertheless *available* to many children. Ecological pairs were as frequent as taxonomic pairs in sort two, and ecological explanations dominated the second sort. Interestingly, both older children and children of all ages from more rural environments were more likely to have ecological relations available to them. These findings suggest two ways in which ecological relations can be learned—both through ambient environment (i.e., living in a more rural area) and through experiences accrued with age.

We examined conceptual flexibility surrounding living things by quantifying change in pairs and explanations from Sort 1 to Sort 2. Results show that conceptual flexibility increased markedly with age, in line with other work (e.g., Blaye et al., 2006; Coni et al., 2019). Notably, living in a more rural environment predicted conceptual flexibility *independently* of age. Thus, children who live in an environment with the potential for environmental exploration not only have ecological relations more available to them, but also were able to flexibly organize plants and animals according to these relations. Surprisingly, results also revealed that children who reported more frequent structured biologically relevant activities, such as visiting aquariums and zoos, demonstrated less flexibility. These patterns may suggest that children who are taught about biology in more structured ways may become entrenched in these taught views and thereby may become less likely to flexibly switch between different types of relations (i.e., Bonawitz et al., 2009; Dane, 2010). This possibility is discussed further below.

Finally, we demonstrated that the salience, but not availability, of ecological relations predicted ecological inferences. Overall, these findings corroborate Shafto et al. (2007b), demonstrating that salience of relations, rather than mere availability, impacts induction. Such findings emphasize the importance of distinguishing between salience and availability, and begin to describe how the ease with which relations come to mind may impact use of concepts in everyday life.

## General Discussion

From a young age, children simplify the complexities of the biological world by constructing intuitive concepts that organize knowledge and guide predictions about plants and animals. While early research on such conceptual development posited a thematic to taxonomic shift, more recent work instead highlights the importance of viewing development as increasing conceptual flexibility. To investigate this, we used unguided card sorting tasks that were designed with two orthogonal dimensions: taxonomic categories (e.g., birds) and ecological relationships (e.g., pond animals). We addressed two sets of research questions. First, we examine developmental changes in the *salience and availability of taxonomic and ecological relations* in children’s intuitive biological thinking. Second, we probe developmental changes in *conceptual flexibility* in the domain of biology (i.e., the ability to shift between taxonomic and ecological thinking about living things). In both cases, we also assessed the impact of environment and activities.

### Salience and Availability of Taxonomic and Ecological Relations

Our first set of questions focused on developmental changes in the *salience and availability of taxonomic and ecological relations* in children’s intuitive biological thinking. Below we consider the implications of our findings for understanding salience, availability, and relations to inductive inference.

#### Salience of Taxonomic and Ecological Relations

We take *salience* to refer to the ease with which knowledge of a particular type of conceptual relation comes to mind or is retrieved; different conceptual relations, even if all are available to an individual, can vary in their salience. In these studies, we took children’s groupings and explanations on the first performance of a sorting task as an index of the salience of taxonomic and ecological relations; to the degree that such relations readily come to mind upon encountering representations of organisms, they should be used to sort and explain. Across age and experience, taxonomic relations were overwhelmingly more salient than ecological relations. In Study 1 and the first sort of Study 2, children were much more likely to pair animals from the same taxonomic category than animals from the same ecosystem, and were much more likely to explain their groupings in taxonomic than in ecological terms. The salience of taxonomic categories was already evident among the youngest participants in our study, and increased with age. This fits with work stressing the importance of richly structured taxonomic categories for children’s understanding of the biological world from early in development (e.g., Scott et al., 1982; Gelman and Markman, 1986; Gelman and Coley, 1990, 1991; Waxman and Namy, 1997; Nguyen, 2007).

As argued above, categories that share rich internal underlying structure also tend to share perceptual and morphological similarities. Likewise, the taxonomic categories we used in the studies presented here, because they were drawn from reality, included members who shared perceptual as well as deep similarities. As such, although we demonstrate a clear early preference for taxonomic categories, our design does not permit us to adjudicate whether this early preference, or indeed any “taxonomic” responses, were based on assumptions of shared rich structure or on perceptual similarity. Although perceptual similarity is nearly always an important guide to richly structured categories, we think there are compelling theoretical (e.g., Murphy and Medin, 1985; Medin, 1989; Ahn et al., 2001) and empirical (e.g., Barsalou, 1983; Gelman and Markman, 1986, 1987; Keil, 1989; Rips, 1989; Heit and Hayes, 2005; Noyes and Keil, 2020) arguments against a strong perceptual view of early category representations. However, the primary aim of the design employed in these studies was to contrast the salience of categories based on intrinsic taxonomic relations–be they perceptual or abstract–with the salience of categories based on extrinsic ecological relations. As such, our results are agnostic with respect to the perceptual-conceptual discussion.

Notably, despite the general salience of taxonomic categories, we also consistently found that the salience of ecological relations was related to environment and experience. In Study 1, although children from rural environments formed as many taxonomic groups as children from urban environments, they were more likely to explain their grouping in ecological terms. In Study 2, children’s environment again did not predict groupings, but the proportion of groups explained in ecological terms increased with children’s unstructured experiences in nature. Whereas the high salience of taxonomic relations further increases with age, ecological relations are more salient among children who have relevant experiences, particularly those who live in rural communities, and those who reported more unstructured nature exploration. This is consistent with a number of studies showing that both relevant activities (e.g., unstructured exploration) and passive experience (i.e., living in more rural environments) increase access to ecological knowledge (e.g., López et al., 1997; Proffitt et al., 2000; Shafto and Coley, 2003), suggesting that salience of thematic relations may be characteristic of expert knowledge (Coley et al., 2005; Coley and Betz, 2018). These and other findings (Medin et al., 1997; Lin and Murphy, 2001; De Deyne et al., 2016) highlight the potency and use of thematic relations in refined conceptual systems.

#### Availability of Ecological Relations

By our account, *availability* refers to whether knowledge of particular conceptual relations is represented in semantic memory, or is readily discernible given the information that is represented in semantic memory. Salient knowledge is necessarily available, but all available knowledge need not (and indeed, cannot) be equally salient. In Study 2, we asked children, after completing their initial sort, to sort the same cards in a different way. We take performance on this second sort as an index of availability of less salient relations; sort 1 allowed children to “discharge” salient knowledge, and sort 2 gave them the opportunity to demonstrate knowledge that, while less salient, was nevertheless available.

Despite being less salient, ecological relations *were* widely available, as indicated by an increase in ecological pairs and an overwhelming preference to provide ecological explanations on sort 2. Notably, we observed a developmental increase in the availability of ecological relations (for explanations). The availability of ecological relations was also consistently related to population density in that–for both pairs and explanations–ecological relations were more available for children from more rural environments. This highlights the potential role of ambient experience in shaping the availability of conceptual relations; in the case of biology, living in an ambient environment with the potential for environmental exploration may encourage the availability of ecological relations.

#### Do Salience and Availability of Conceptual Relations Predict Patterns of Inductive Inference?

By comparing the sorting performance of children in Study 2 to their performance on a previously published triad induction task (Coley, 2012), we also asked whether the salience and/or availability of taxonomic and ecological relations influence patterns of inductive reasoning. Results clearly showed that children for whom ecological relations were more salient (both pairs and explanations) were also more likely to make ecological inferences (i.e., project a novel property to an ecologically related species rather than a taxonomically related species). In contrast, the mere availability of conceptual relations did not predict inductive preferences. Thus, the ease with which conceptual relations come to mind (i.e., salience), but not the mere presence of such relations in one’s conceptual toolbox (i.e., availability) influences the recruitment of such relations when making predictions about the world.

Results also illuminate previous findings on the development of selectivity in inductive inference. Coley (2012) reported that rural children were precociously sensitive to context on the triad induction task described here, with 6-year-old rural participants making more taxonomic inductions when reasoning about *insides*, and more ecological inductions when reasoning about *diseases*. In contrast, urban and suburban children did not show evidence of this context sensitivity until later ages (10 and 8 years, respectively). The current findings shed light on a potential mechanism behind this context sensitivity: heightened baseline salience of ecological knowledge for rural children. Heightened salience of ecological relations might allow rural children to access either taxonomic or ecological knowledge in the context of inductive inference depending on the property being projected. In contrast, ecological knowledge in urban children—even if available—might not be sufficiently salient to allow differential access among younger urban participants.

#### Salience and Availability as Distinct Constructs

Our results support the idea that the distinction between salience and availability of conceptual relations is a useful one for understanding conceptual development. This distinction is highlighted by performance on the sorting task. There, salience of ecological relations was predicted by children’s unstructured activities; in contrast, availability of ecological relations was predicted by rural environment. Thus, active engagement predicted salience whereas living in an environment that provides opportunities for passive nature observation predicted availability. Moreover, salience of relations predicted patterns of inductive inference, whereas availability was unrelated to inferences. Thus, it seems like considering the salience and availability of conceptual relations separately contributes to our understanding of development. These findings build on and expand ideas about availability in inductive reasoning (Shafto et al., 2007b).

### Conceptual Flexibility

We posit that to demonstrate conceptual flexibility within a domain, an individual must represent multiple relevant conceptual relations, and also be able to inhibit salient relations and flexibly access and use other available knowledge. As such, it represents both domain specific knowledge and domain-general executive functions. Our findings fit with this dual characterization. We defined conceptual flexibility within our study as the extent to which children changed their reliance on both types of predicted organizational relations (i.e., ecological and taxonomic) across sorts one and two. Not only did we observe strong and consistent increases in conceptual flexibility with age, we also saw increased conceptual flexibility in the domain of biology among children living in more rural areas. This suggests that development of conceptual flexibility is driven by domain-specific experience as well as the emergence of domain-general executive functions.

Results clearly showed a marked increase in conceptual flexibility with development, for both pairs and explanations. This could represent the contribution of development of domain-general executive functions like cognitive flexibility and inhibitory control, and/or the acquisition of taxonomic and ecological knowledge via formal or informal means. Executive functions–typically seen as working memory/information updating, cognitive flexibility/shifting, and inhibitory control (Miyake et al., 2000)–are defined as “adaptive, goal-directed behaviors that enable individuals to override more automatic or established thoughts and responses (Garon et al., 2008, p. 31).” Although basic components are typically in place before children begin formal education (Garon et al., 2008), executive functions like inhibitory control and working memory undergo a gradual developmental trajectory that extend well into late adolescence and young adulthood (Davidson et al., 2006; Diamond, 2013). The increase with age that we observed in conceptual flexibility undoubtedly reflects in part an increase in general executive function being demonstrated in intuitive biological thought. This view fits with that of Spiro (1988), who argued that individuals shift from a single representation to multiple representations, which acts as a foundation for cognitive flexibility. Similarly, Deak (2003) suggested that individuals first create a range of plausible ways to organize information and that flexibility allows them to select appropriate patterns based on task demands.

The finding of increased conceptual flexibility in the domain of biology among children living in more rural areas suggests an impact of domain-specific experience on the development of conceptual flexibility. Living in rural environments, and having the opportunity to directly observe and interact with species in relatively intact ecosystems, could facilitate the development of conceptual flexibility in a number of ways. Most simply, such experience might provide children living in rural environments with knowledge of ecological relations among species; children living in more urban environments might not have access to such knowledge. In order to demonstrate conceptual flexibility, a child must have multiple conceptual systems available. Perhaps rural children are more flexible because they possess knowledge about ecological relations among living things that they can demonstrate on a second sort, whereas children living in urban environments typically lack such knowledge. One piece of evidence suggesting that this difference cannot be attributed solely to differential knowledge is the negative relationship between the “structured activities” factor and conceptual flexibility. One might expect children who tend to frequent zoos and museums to possess a relatively large reservoir of factual knowledge, but if so, this isn’t sufficient to guarantee conceptual flexibility. Another possibility is that more varied experience with living things–such as that afforded by living in a less densely populated locale–facilitates the development of more general executive function capacities. Previously demonstrated linkages between individual differences in the development of theory-like intuitive biological thinking and executive function have been interpreted to show that executive function provides a foundation for the development of intuitive biology (Zaitchik et al., 2014). However, analogous to claims about effects of bilingualism on executive function (e.g., Bialystok and Martin, 2004; Bialystok et al., 2014), perhaps living in a relatively rich biological environment scaffolds the development of executive functions more generally by requiring increased inhibitory control and cognitive flexibility in children as they negotiate their surroundings and construct representations of kinds and relations therein. Our results cannot arbitrate between these two possibilities, but they raise important questions for future research.

Thus, these results support those of Coley (2012), and other work showing an increase in the flexible use of multiple conceptual dimensions among experts (e.g., López et al., 1997; Shafto and Coley, 2003; see Shafto et al., 2007b, for a review). It is important to emphasize that the increase in conceptual flexibility with decreasing population density was specific to intuitive biological thinking. We did not include any general measure of executive function, and moreover, we would hypothesize that any developmental context providing the opportunity for children to observe and interact with complexity would give rise to increased conceptual flexibility in that domain. For example, children growing up in urban settings might show greater conceptual flexibility with respect to social categories, or spatial wayfinding. Nevertheless, these findings raise the possibility that the acquisition of domain specific knowledge or experience could bolster a general cognitive ability and may suggest an important domain-specific component to executive function.

### Conceptual Development as Increasingly Flexible Use of Multiple Relations

Our results are more consistent with a view of development as increasing conceptual flexibility than a shift from emphasis on one dimension to another. When looking only at salience of taxonomic and ecological relations, our findings align somewhat with the notion of a thematic to taxonomic shift; taxonomic relations became increasingly salient across development, while *salience* of ecological relations decreased across development. However, counter to the idea that knowledge of taxonomic relations emerges later in development, the responses of even our youngest participants were overwhelmingly taxonomic. Moreover, the availability of ecological relations increased with development and with experience. The early salience of taxonomic relations and increasing availability of ecological relations are inconsistent with the idea that young children prefer thematic relations, and that reliance on them decreases with age. These, along with the fact that conceptual flexibility increased markedly with age, suggest instead that development is more profitably characterized as the increasing emergence of the ability to flexibly utilize knowledge of different relations for different purposes (see also Gelman and Markman, 1986; Waxman and Namy, 1997; Ross and Murphy, 1999; Thibaut et al., 2016; for a review, see Lin and Murphy, 2001).

### Implications for Science Education

#### Informal Experience Facilitates Conceptual Flexibility

Conceptual flexibility is critical in learning biological science as well as other STEM fields. In biology, one must be able to think about biological entities and concepts at different levels of analysis. Indeed, this is a guiding principle of the “Vision and Change” core concepts in undergraduate biology education (American Association for the Advancement of Science, 2011) as well as the “crosscutting concepts” in the *Next Generation Science Standards* for K-12 education (National Research Council, 2012). As such, demonstrating conceptual flexibility might be a signal that a child is beginning to ‘think like a scientist’ (e.g., Williams et al., 2004). Indeed, formal scientific thought requires the ability to hold and flexibly navigate between multiple different types of relations. Our findings highlight the benefits of informal domain-specific experiences (e.g., Ritter et al., 2012) in developing domain-specific cognitive flexibility. We have shown that simultaneously understanding that organisms can belong to ecological as well as taxonomic groups increases with age, and moreover that the environment in which a child lives explains independent variance in such conceptual flexibility. Thus, children’s informal experience impacts the development of conceptual systems that form an important basis for learning science and affects the degree to which children come into the classroom with an ability to think flexibly about relations among organisms.

#### Informal Experience Can Impede Conceptual Flexibility

While experience can facilitate cognitive flexibility, there also may be a trade-off between the potential outcomes of flexibility and *cognitive entrenchment*—a high level of stability in thinking that can come with expertise or formal education (for a review, see Dane, 2010). Consistent with this idea, we found that frequent participation in more structured (albeit informal) activities, such as visiting aquariums and zoos, predicted less conceptual flexibility. This somewhat counterintuitive relationship between experience and rigidity in thinking aligns with ideas of a ‘double-edged sword of pedagogy’ (e.g., Bonawitz et al., 2009, 2011) wherein formal instruction not only benefits children’s ability to achieve a targeted goal (e.g., successfully making one toy’s function work) but also decreases their likelihood to creatively explore (e.g., find other functions of the toy). Structured experiences that are clearly meant to convey information can help children learn more quickly but can also discourage explorative play by suggesting that there are ‘right’ and ‘wrong’ ways of thinking about a domain, or that the important information has already been conveyed. To some extent, this could be quite beneficial; it could help individuals converge on more conventional ways of organizing their knowledge, which could facilitate communication and could help individuals focus on relations that are known to have robust inductive utility. At the same time, this could also restrict individuals’ creativity and novel interpretations of the world, which might inhibit conceptual flexibility. One factor that could encourage entrenchment at the expense of flexibility might be the lack of engagement with a “dynamic environment within one’s expertise domain” (Dane, 2010; p. 589). Together with the theory of pedagogy’s ‘double-edged sword,’ this might elucidate the asymmetry that we found between effects of rural environment (associated with more conceptual flexibility) and structured activities (associated with less conceptual flexibility); dynamic, diverse, and perhaps unguided experiences might help encourage more flexible thinking.

### Future Research

These findings raise important avenues for future research both to enhance the field’s understanding of conceptual development and to bolster science education. First and foremost, it will be critical to determine how children transition from having multiple relations available to being able to flexibly shift between these relations in real time. To answer this question, it will be useful to describe the interactions between developing domain-specific conceptual flexibility and more domain-general executive functions. While we have speculated that executive functions such as working memory or inhibitory control are important for conceptual flexibility, our results do not directly speak to their interactions. Finding this predicted relationship would have implications for age-related limitations in conceptual development, and would suggest potential interventions to improve conceptual flexibility.

Another important next step involves investigating the role of conceptual flexibility in the biology classroom. Of particular interest is whether increased salience or availability of ecological relations, or increased conceptual flexibility surrounding biological entities, predicts academic success. Such findings would demonstrate the importance of intuitive biology beyond our daily interactions with plants and animals, demonstrating how these informal ways of learning can provide a leg up in school. Likewise, research should also investigate the effects of both structured and unstructured activities in conceptual flexibility. Our findings provide preliminary correlational evidence that both types of activities could factor into a potential trade-off between conceptual flexibility and cognitive entrenchment. Delving deeper into this relationship could help identify a proper amount of structure in activities that provide children with information quickly, but also encourage them to explore and discover some of the answers on their own. This type of activity could not only enhance learning but also create positive attitudes of self-efficacy surrounding knowledge acquisition.

### Limitations

As noted above, our study is limited in its ability to explain why children formed taxonomic categories—on the basis of perceptual similarities, due to richly structured representations shared underlying features, or a combination thereof. However, it was never our goal to investigate the basis of taxonomic categorization *per se*, but rather to demonstrate that children are able to flexibly switch between two types of salient categories—taxonomic and ecological—and to elucidate the role of experience in the development of this ability. Therefore, we do not believe that this limitation compromises our conclusions about the development of conceptual flexibility.

Relatedly, our study is limited to some extent by the stimuli that we selected. Some of our taxonomic groups share more functional and perceptual similarities (e.g., the wings of the insects) than others (e.g., the more diversified sets of plants). These stimuli choices could have influenced the types of categories that participants generated. For example, the perceptual similarities in some of the taxonomic categories in our card set could have cued certain patterns of taxonomic groupings that could overestimate the salience of taxonomic relations among sampled children. Further, there are also ecological relations that children may know about that we did not probe within the card sets. This could have led to an underestimation of the salience of ecological relations among the sampled children.

Finally, we only included stimuli featuring local animals within our stimuli set because we expected direct experience with local animals to be relevant to ecological thinking. Thus, we are unable to determine whether the differences we observed between children from rural versus urban environments would generalize to reasoning about unfamiliar or exotic species. On one hand, if ecological relations are more broadly salient to rural children, we might expect rural children to still produce more ecological relations for more exotic species. That is, their experience might increase the salience of ecological relations in general, and therefore transfer to contexts beyond their immediate experience. On the other hand, if the salience of ecological relations specifically stems from experience with the local species, and perhaps the opportunity to observe those relations directly, this salience may not generalize to more exotic creatures. In this case, we would expect rural and urban children to perform similarity for unfamiliar or exotic species. This question gets at more general issues of knowledge transfer, and is therefore an important avenue for future research.

## Conclusion

Using an open-ended sorting task, we have shown that taxonomic relations among living things are overwhelmingly salient to children independent of age, environment, and experience. However, by distinguishing between the salience and the availability of conceptual relations, we also show that despite this general salience of taxonomic relations, the availability of ecological relations increased with age and by both direct experience with nature (i.e., through informal exploration) and the population density of their hometown. Critically, we showed that conceptual flexibility–the ability to switch between thinking about organisms taxonomically and ecologically–increases with age but is also predicted by experience. We argue that these results point to an account of conceptual development as the emergence of flexible multidimensional conceptual systems for thinking about domains of experience, as exemplified here in the domain of biology. Finally, our results document the importance of taking children’s background into account in science teaching and learning, by demonstrating that life experiences—what children bring to the science classroom—can impact both domain specific and domain general cognitive processes critical for the development of scientific thought.

## Data Availability Statement

The datasets generated for this study are available on request to the corresponding author.

## Ethics Statement

The studies involving human participants were reviewed and approved by Northeastern University Institutional Review Board. Written informed consent to participate in this study was provided by the participants’ legal guardian.

## Author Contributions

JC designed the study, collected the data, and assisted with data analysis and writing of the manuscript. NB led the data analysis and writing of the manuscript. Both authors contributed to the article and approved the submitted version.

## Conflict of Interest

The authors declare that the research was conducted in the absence of any commercial or financial relationships that could be construed as a potential conflict of interest.
