# Supplementary material for: Development of Conceptual Flexibility in Intuitive Biology: Effects of Environment and Experience
Source: Front Psychol. 2020 Sep 16;11:537672. doi: 10.3389/fpsyg.2020.537672 (PMC7525208; doi:10.3389/fpsyg.2020.537672)
Supplement: Supplementary file 1 [file Data_Sheet_1.docx]

**Supplementary Materials**

Development of Conceptual Flexibility in Intuitive Biology: Effects of Environment and Experience

Nicole Betz & John D. Coley

**Table of Contents**

**Study 2 Explanations: Detailed Coding Process,** Pages 2-3

***Table 1*.** Study 2 Explanations: Example Codes and Prevalence in Sorts 1 and 2

**Study 2 Percentage of Taxonomic Percentage Pairs, Sorts 1 and 2,** Pages 4-6

**Figure 1**. Histograms Depicting Percentages of Sort 1 Taxonomic and Ecological
Percentage Pairs, Study 2

**Figure 2.** Histograms Depicting Percentages of Sort 1 Taxonomic and Ecological Explanations, Study 2

**Figure 3.** Histograms Depicting Percentages of Sort 2 Taxonomic and Ecological Percentage Pairs, Study 2

**Figure 4**. Histograms Depicting Percentages of Sort 2 Taxonomic and Ecological Explanations, Study 2

**Study 2 Performance between Sorting and Triad Task Specific Induction Properties**, Pages 6-7

***Table 2****.* Relations between Indices of Performance on Sorting Task and Preferences for Ecological Inferences in the Triad Induction Task: Diseases

***Table 3****.* Relations between Indices of Performance on Sorting Task and Preferences for Ecological Inferences in the Triad Induction Task: Insides

**Study 2 Explanations: Detailed Coding Process**

Explanations for Study 2 were initially coded by a team of 3-4 trained coders using a constant comparative method (Glaser and Strauss, 1967, Boeije, 2002). Explanations were initially categorized into one of the following ten categories that were determined a priori based on their relationships to taxonomic and ecological relationships and based on Coley & Vasilyeva (2010): a) ***taxonomy*** (i.e., explicitly mentioning that the organisms belonged to the same taxonomic groups or were the same ‘kind’ of animal), b) ***general interaction*** (i.e., mentioning that the organisms within the groups interacted with one another in an unspecified way), c) ***diet interaction*** (i.e., explicitly mentioning that the animals interacted because they had a predator/prey relationship), d) ***habitat interaction*** (i.e., explicitly mentioning that the organisms within the groups interacted with one another because of their shared habitat), e) ***similar habitat*** (i.e., explicitly mentioning that the organisms within the group live in the same habitat), f) ***similar diet*** (i.e., explicitly mentioning that the organisms within the eat the same food), g) ***similar behavior*** (i.e., justifying the group based on the organisms’ ability to engage in similar behaviors), h) ***similar appearance*** (i.e., mentioning that the organisms were grouped together due to shared physical features), i) ***general similarity*** (i.e., justifying groups based on the fact that the organisms in the group were ‘similar’ to one another), and j) ***other*** (i.e., any justification that did not fit into these other categories. For sample explanations and prevalence of each of these types of codes, see Table 1.

Based both on a priori predictions and a review of the content of the justifications within each of the specific codes, eight of the ten categories were merged into two overarching categories of ‘Taxonomic and ‘Ecology.’ The Taxonomic overarching category consisted of only one preliminary category: taxonomy.The Ecology overarching category consisted of the following five initial categories: general interaction, diet interaction, habitat interaction, similar habitat, and similar diet.

***Table 1*.** Study 2 Explanations: Example Codes and Prevalence in Sorts 1 and 2.

| **Ultimate Code** | **Preliminary Code** | **Example Explanations** | **Prevalence**  **(Sort 1)** | **Prevalence**  **(Sort 2)** | ***t*-test** |
| --- | --- | --- | --- | --- | --- |
| *Taxonomy* | *Taxonomy* | *"birds", "insects", "plants"* | 57.88% | 16.18% | **16.27***** |
| *Taxonomy* | *General Similarity* | *"similar", "they are all the same"* | 5.48% | 4.52% | 1.10 |
|  |  |  |  |  |  |
| *Ecology* | *General Interaction* | *"both like trees", "maybe they are best friends"* | 2.68% | 6.97% | **-4.15***** |
| *Ecology* | *Diet Interaction* | *"ducks eat cattail near water", "eat grasshoppers"* | 4.84% | 15.86% | **-7.31***** |
| *Ecology* | *Habitat Interaction* | *"woodpeckers and bluejays live in trees",*  *"saw slug on pine tree"* | 4.17% | 9.69% | **-4.02***** |
| *Ecology* | *Similar Habitat* | *"usually on the edge of the path by the ocean", "birds that live in trees"* | 7.10% | 12.87% | **-3.75***** |
| *Ecology* | *Similar Diet* | *"eat wood", "grasshopper likes to eat leaves so does termites"* | 1.07% | 1.86% | -1.25 |
|  |  |  |  |  |  |
| *Neither* | *Similar Appearance* | *"slimy animals", "same color", "look alike"* | 10.00% | 9.22% | .479 |
|  |  |  |  |  |  |
| *Neither* | *General Similarity* | *"similar", "they are all the same"* | 5.48% | 4.52% | 1.10 |
|  |  |  |  |  |  |
| *Neither* | *Similar Behavior* | *"slithers", "jumps far", "migrate"* | 9.52% | 13.81% | **-2.79**** |
| *Neither* | *Other* | *"doesn't go [with others]", "nothing goes with it", "excellent"* | 8.19% | 8.98% | .77 |
|  |  |  |  |  |  |

Note: the final column on the right reports t-test statistic for comparisons between each preliminary code across the two sorts. **p<.01; ***p<.001.

**Study 2 Percentage of Taxonomic Percentage Pairs, Sorts 1 and 2**

Our measure of conceptual flexibility assessed the extent to which children changed the underlying dimension around which they organized their two sorts between taxonomic and ecological relations. One criterion which allowed us to justify this metric was the fact that one of the two types of relations most often strongly dominated the sorts, suggesting that children often did tend to use one type of relation to organize their sorts—either taxonomic or ecological. This trend is most evident in Sorts 1 (see Figures 1 and 2), where the histograms depicting percentage of pairs and explanations show that children predominantly organized Sort 1 based on taxonomy. The Sort 2 graphs (Figures 3 and 4) show that children largely did not sort by taxonomic relations in Sort 2, but were mixed in the extent to which they relied in ecological relations. Overall, these figures reveal that children did indeed tend to rely on one of the two a prior overarching frameworks as an underlying framework for their sorts.


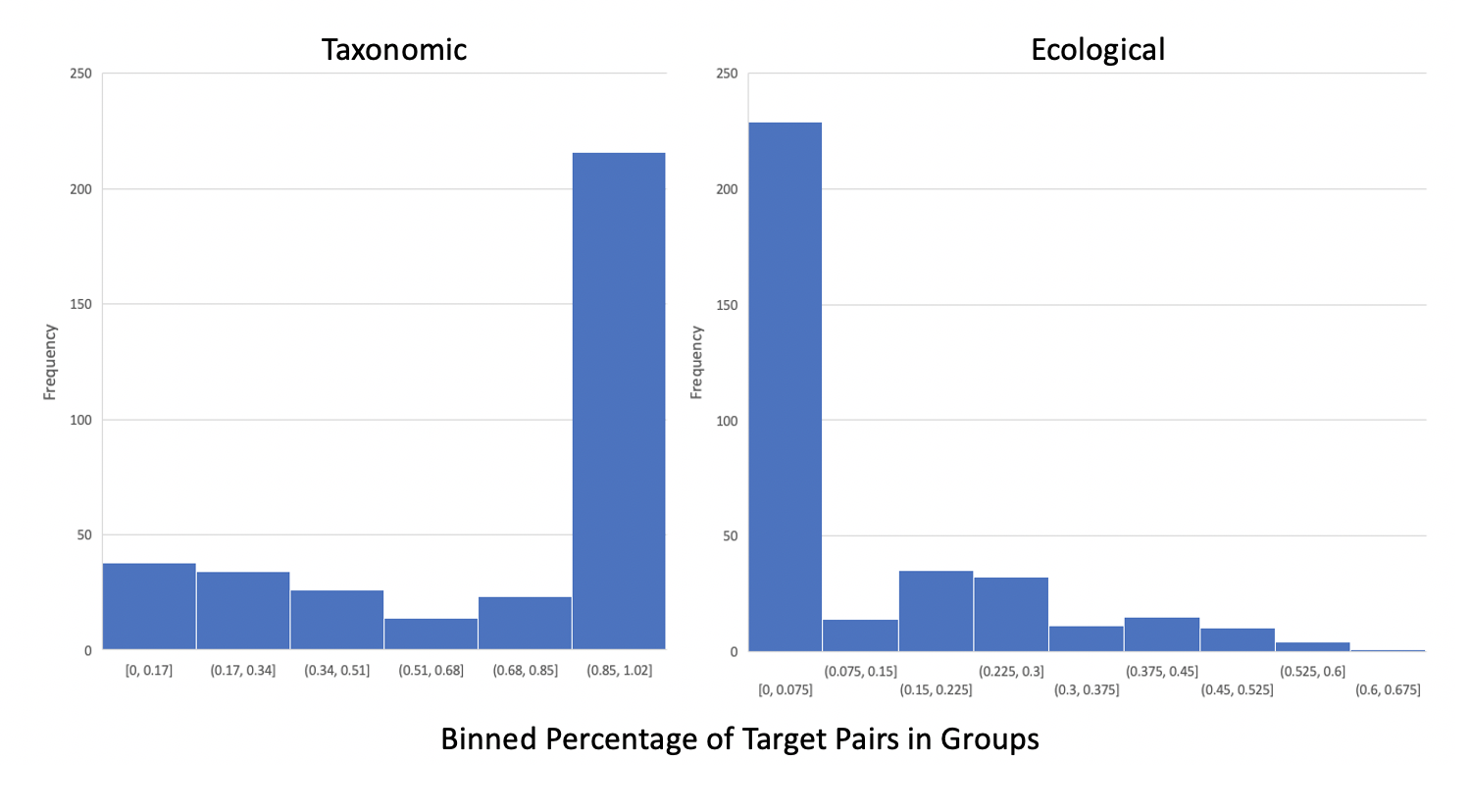


**Figure 1**. Histograms Depicting Percentages of Sort 1 Taxonomic and Ecological
Percentage Pairs, Study 2


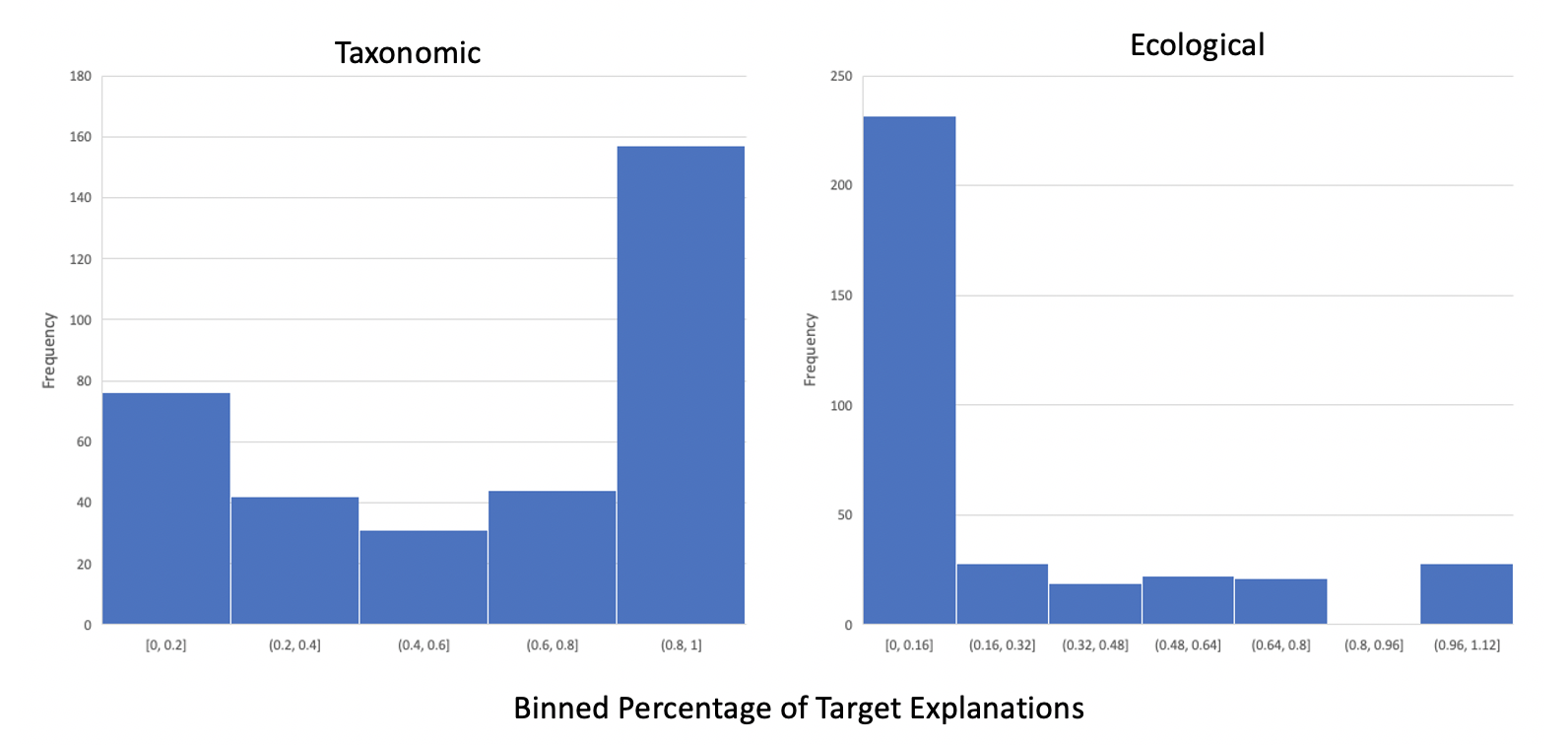


**Figure 2.** Histograms Depicting Percentages of Sort 1 Taxonomic and Ecological Explanations, Study 2


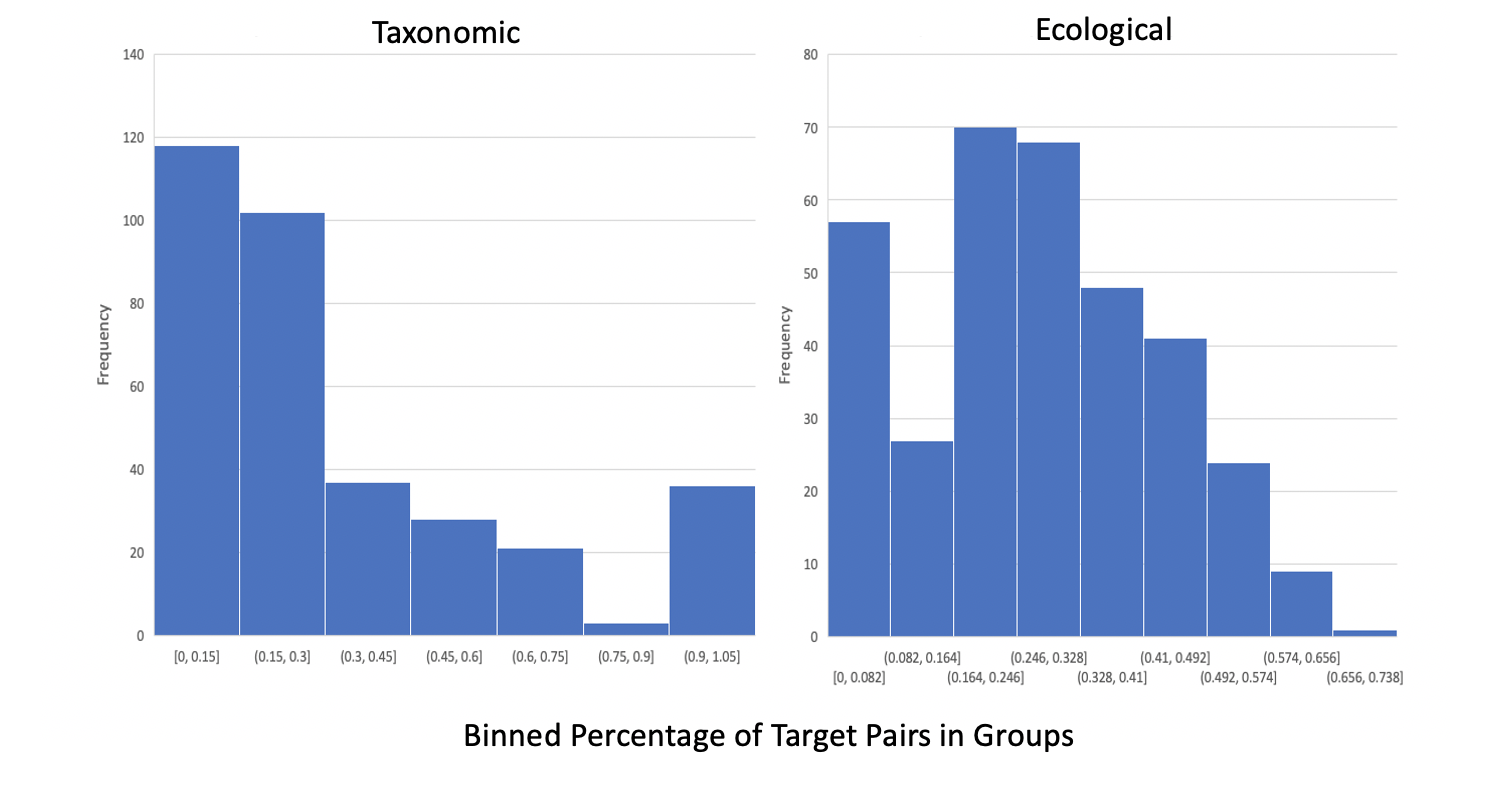


**Figure 3.** Histograms Depicting Percentages of Sort 2 Taxonomic and Ecological Percentage Pairs, Study 2


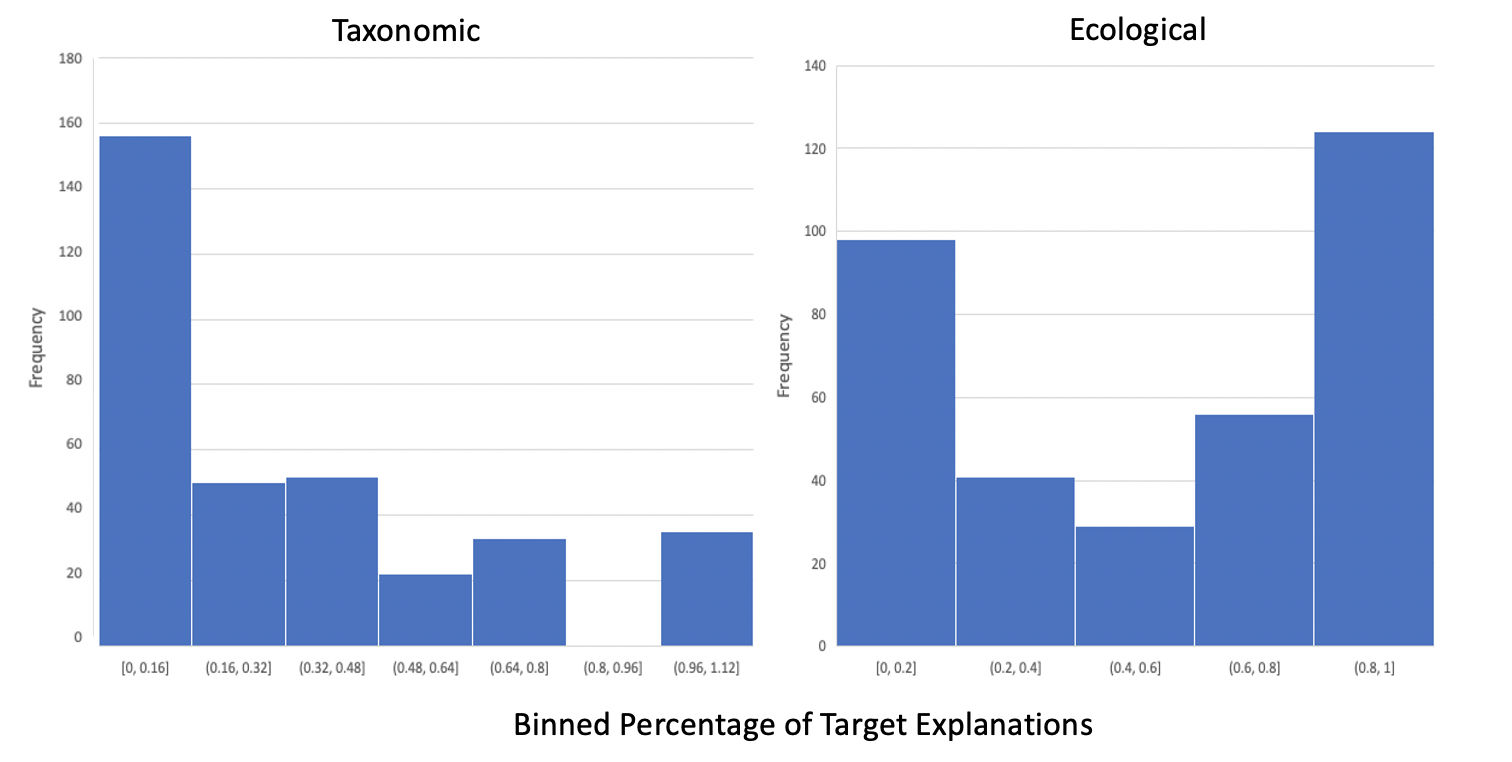


**Figure 4**. Histograms Depicting Percentages of Sort 2 Taxonomic and Ecological Explanations, Study 2

**Study 2 Performance between Sorting and Triad Task Specific Induction Properties**

Within the triad task, children were given one of two types of properties on which to make inferences—either diseases or insides. For simplification, data were collapsed across property type in the main text. Below, we report relations between performance on the sorting task and triad tasks for these two specific induction properties.

To examine relations between children’s sorting and their inductive inferences, we ran separate regressions using children’s first and second sort taxonomic and ecological pairs and explanation to predict their proportion of ecological inferences on the triad induction task (i.e., the percentage of trials a child projected the novel property to the ecological target rather than the taxonomic target). Each measure of performance was entered into a separate regression, along with age and log population density as control variables (both of which have been linked to ecological inferences, Coley 2012). Results for children who reasoned about diseases within the triad task are reported in Table 2, and results for children who reasoned about insides are reported in Table 3.

Across the two types of specific properties, regressions revealed two significant positive predictors of ecological inferences. When reasoning about shared diseases, the only significant predictor was percentage of ecological explanations in the first sort. When reasoning about shared insides, the only significant predictor was the percentage of ecological pairs in the first sort. Further, we also found that percentage of taxonomic pairs in the first sort was a marginal negative predictor for ecological inductions about shared insides. Overall, these findings reveal that triad inductions were linked to salience of ecological relations.

***Table 2****.* Relations between Indices of Performance on Sorting Task and Preferences for Ecological Inferences in the Triad Induction Task: Diseases

| **Sort** | **Relation Type** | **Performance Index** | **Standardized Regression Coefficient** |
| --- | --- | --- | --- |
| **1** | ***Ecological*** | *% Pairs* | .119 |
|  |  | *% Explanations* | **.181*** |
|  | ***Taxonomic*** | *% Pairs* | -.088 |
|  |  | *% Explanations* | .016 |
| **2** | ***Ecological*** | *% Pairs* | .040 |
|  |  | *% Explanations* | .121 |
|  | ***Taxonomic*** | *% Pairs* | .038 |
|  |  | *% Explanations* | -.038 |

Note: **p*<.05

***Table 3****.* Relations between Indices of Performance on Sorting Task and Preferences for Ecological Inferences in the Triad Induction Task: Insides

| **Sort** | **Relation Type** | **Performance Index** | **Standardized Regression Coefficient** |
| --- | --- | --- | --- |
| **1** | ***Ecological*** | *% Pairs* | **.171*** |
|  |  | *% Explanations* | .074 |
|  | ***Taxonomic*** | *% Pairs* | **-.145+** |
|  |  | *% Explanations* | -.079 |
| **2** | ***Ecological*** | *% Pairs* | -.022 |
|  |  | *% Explanations* | -.001 |
|  | ***Taxonomic*** | *% Pairs* | .060 |
|  |  | *% Explanations* | -.013 |

Note: +p<.10; **p*<.05
